# Supplementary material for: Pectins Rich in RG-I Extracted from Watermelon Peel: Physicochemical, Structural, Emulsifying, and Antioxidant Properties
Source: Foods. 2024 Jul 25;13(15):2338. doi: 10.3390/foods13152338 (PMC11311835; doi:10.3390/foods13152338)
Supplement: Supplementary file 1 [file foods-13-02338-s001.zip › supplement.pdf]

Table S1

Linkage patterns and corresponding percentages of RG-I enriched pectic polysaccharides were extracted from watermelon peel (RPWP)

RT	Methylated Sugar	Type of Linkage	Molar Ratio	Deduced Polymers
	Xylose		***3.1%***	
15.51	2,3-Me_2-Xylp	→4)-Xylp-(1→	3.1%	Xylan
	Arabinose		***24.9%***	
15.27	2,3-Me_2-Araf	→5)-Araf-(1→	3.7%	RG-I (AG-I)
19.06	2-Me_1-Araf	→3,5)-Araf-(1→	5.0%	RG-I(AG-I)
10.55	2,3,5-Me_3-Araf	Araf-(1→	16.2%	RG-I (AG- I/II)
	Rhamnose		***5.5%***	
13.96	2-Me_1-Rhap	→3,4)-Rhap-(1→	2.8%	RG-I
19.15	3-Me_1-Rhap	→2,4)-Rhap-(1→	2.7%	RG-I
	Glucose		***3.4%***	
17.02	2,3,4,6-Me_4-Glcp	Glcp-(1→	1.5%	Other
27.19	2,4-Me_2-Glcp	→3,6)-Glcp-(1→	1.9%	Other
	Galactose/ Galacturonic acid		***63.1%***	
21.89	2,4,6-Me_3-Galp	→3)-Galp-(1→	3.2%	RG-I (AG-II)
28.70	2,4-Me_2-Galp	→3,6)-Galp-(1→	12.8%	RG-I (AG-II)
17.97	2,3,4,6-Me_4-Galp	Galp-(1→	9.5%	RG-I/ HG
23.81	2,3,4-Me_3-Galp	→6)-Galp-(1→	1.7%	RG-I (AG-II)
24.46	2,6-Me_2-Galp	→3,4)-Galp-(1→	1.7%	RG-I (AG-II)
21.21	2,3,6-Me_3-Galp	→4)-Galp-(1→	34.2%	HG/ RG-I

calculated as a relative peak area percentage of all alditol acetate derivatives present. HG: homogalacturonan. AG-I/II: arabinogalactan I/II. RG-I: rhamnogalacturonan I

Table S2

Chemical shifts of monosaccharide residues in RG-I enriched pectic polysaccharides were extracted from watermelon peel (RPWP) from the ^{1}H-^{13}C NMR spectrum

Residue	Chemical Shift, δ (ppm)					
	H-1<br>C-1	H-2<br>C-2	H-3<br>C-3	H-4<br>C-4	H-5<br>C-5	H-6<br>C-6
→5)-Araf-(1→	5.09<br>109.25	4.10<br>83.32	4.03<br>79.08	4.19<br>85.31	3.83<br>69.27	
→3,5)-Araf-(1→	5.04<br>106.18	4.24<br>82.75	4.08<br>84.86	3.88<br>83.05	3.93<br>69.27	
Araf-(1→	5.07<br>109.25	4.09<br>80.94	4.05<br>76.43	3.73<br>67.54	3.96<br>63.33	
→3,4)-Rhap-(1→	5.18<br>98.68	4.18	5.2<br>74.99	3.89<br>76.59		
→2,4)-Rhap-(1→	4.96<br>99.46	4.10<br>77.57	4.05<br>74.93	3.88<br>83.41	3.79<br>66.48	1.11<br>16.78
→3)-Galp-(1→	4.61<br>104.32	4.0<br>77.0	3.85<br>65.73	4.11<br>77.4	4.21<br>79.88	3.91<br>60.99
→3,6)-Galp-(1→	4.58<br>106.18	3.51<br>72.16	3.69<br>82.91	4.10<br>70.99	3.94<br>76.72	3.93<br>69.21
Galp-(1→	5.04<br>108.42	4.07<br>70.21	3.85<br>65.28	4.18<br>75.37	4.21<br>73.78	3.91<br>60.87
→4)-GalpA-(1→	4.96<br>101.63	3.52<br>66.23	3.84<br>67.76	4.23<br>79.28	4.69<br>70.21	172.57

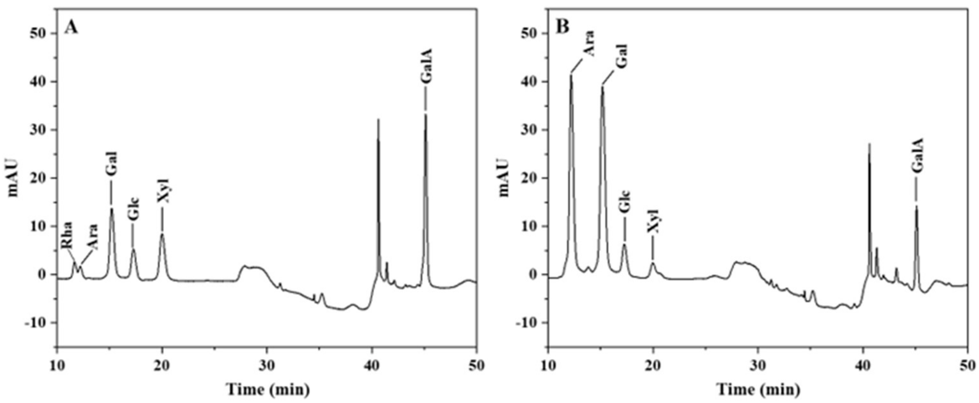

Figure. S1 Monosaccharide compositions of commercial pectin (CP) (A) and RG-I enriched pectic polysaccharides were extracted from watermelon peel (RPWP) (B).

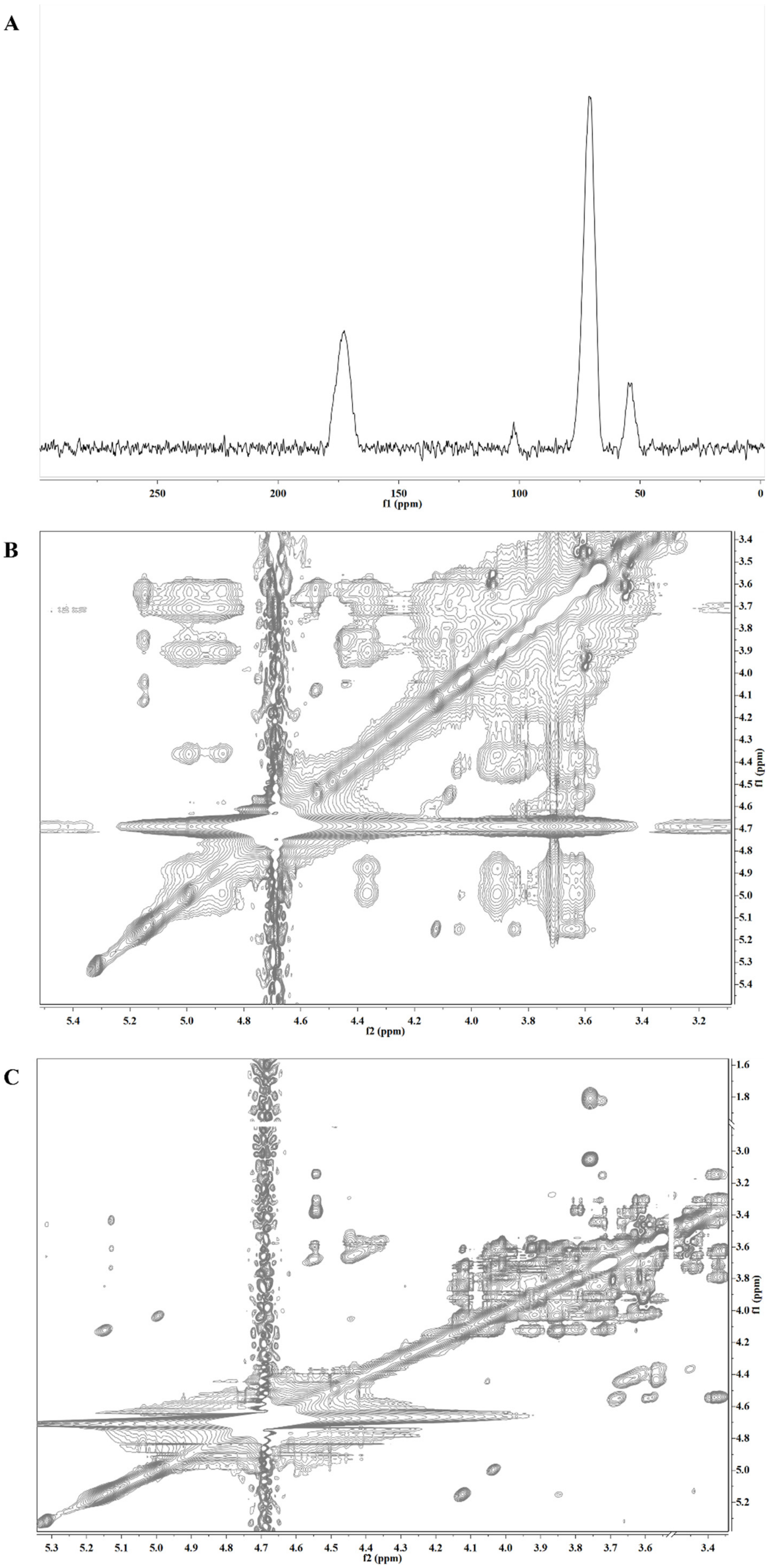

A
250
200
150
100
50
0
f1 (ppm)
B
f2 (ppm)
f1 (ppm)
C
f2 (ppm)
f1 (ppm)

Figure. S2 ^{13}C nuclear magnetic resonance (NMR) spectrum (A), NOESY NMR spectrum (B) and TCOSY NMR spectrum (C) for RG-I enriched pectic polysaccharides were extracted from watermelon peel (RPWP) RG-I enriched pectic polysaccharides were extracted from watermelon peel (RPWP).
