# Supplementary figures and images for: Pectins Rich in RG-I Extracted from Watermelon Peel: Physicochemical, Structural, Emulsifying, and Antioxidant Properties
Source: Foods. 2024 Jul 25;13(15):2338. doi: 10.3390/foods13152338 (PMC11311835; doi:10.3390/foods13152338)

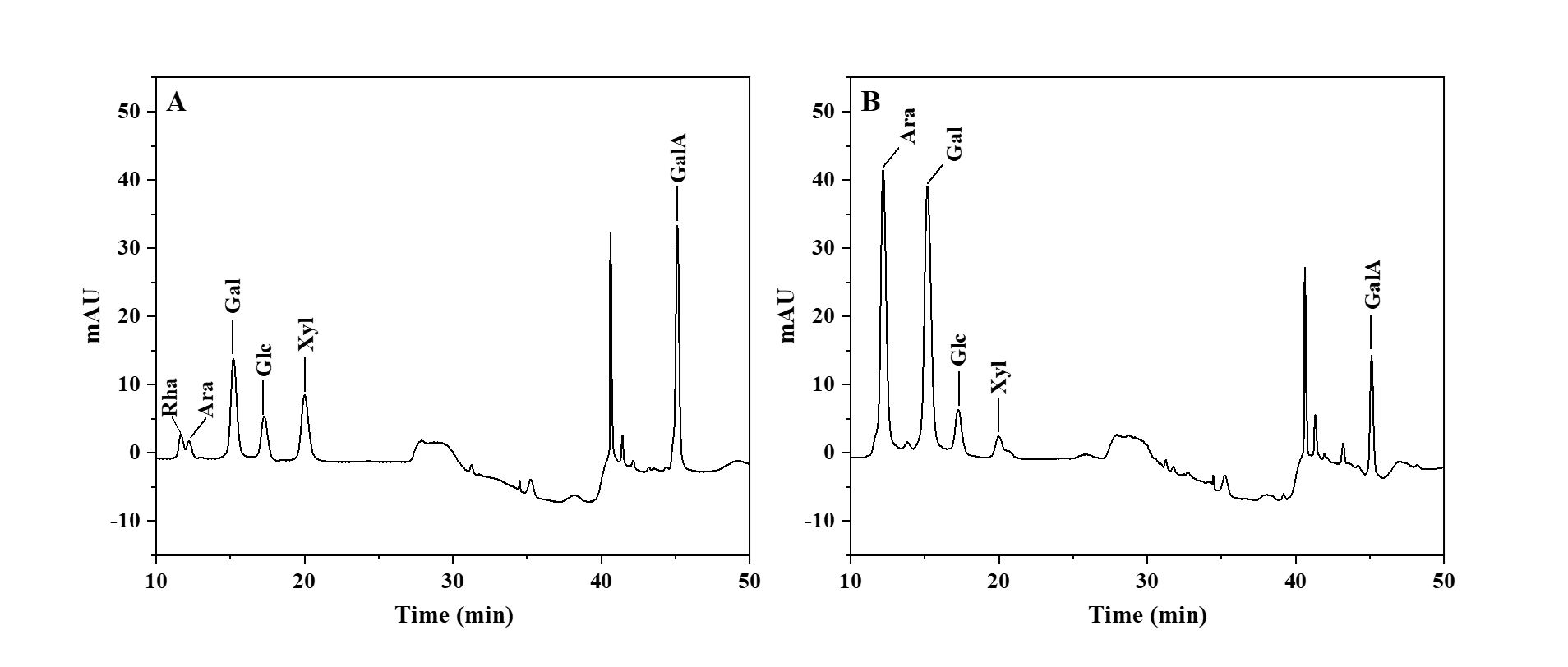

Supplement: Supplementary file 1 [file foods-13-02338-s001.zip › Figure S1.tif]

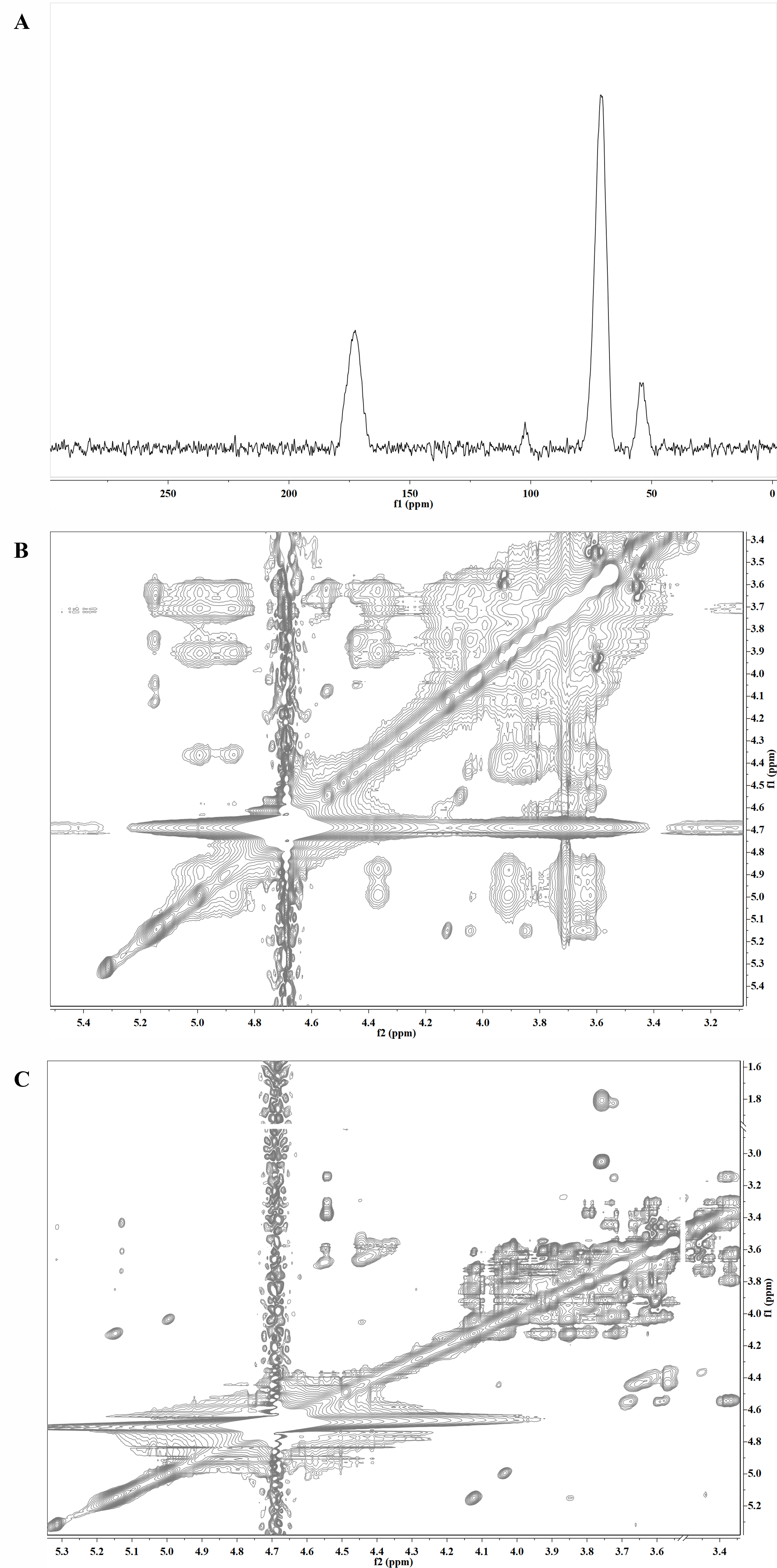

Supplement: Supplementary file 1 [file foods-13-02338-s001.zip › Figure S2.tif]
